# Skeletal muscle loss in the postoperative acute phase after esophageal cancer surgery as a new prognostic factor

**DOI:** 10.1186/s12957-020-01908-6

**Published:** 2020-06-26

**Authors:** Naoaki Maeda, Yasuhiro Shirakawa, Shunsuke Tanabe, Kazufumi Sakurama, Kazuhiro Noma, Toshiyoshi Fujiwara

**Affiliations:** Department of Gastroenterological Surgery, Graduate School of Medicine, Dentistry and Pharmaceutical Sciences, Okayama University, 2-5-1 Shikatacho, Kita-ku, Okayama, 700-8558 Japan

## Abstract

**Background:**

The postoperative survival rate of patients with esophageal squamous cell carcinoma (ESCC) remains poor compared with other gastrointestinal cancers. We hypothesized that skeletal muscle loss in the postoperative acute phase might be a new predictor for long-term prognosis after highly invasive surgery such as ESCC surgery.

**Methods:**

The following items were retrospectively investigated. First, whether skeletal muscle loss occurred in the postoperative acute phase of ESCC was verified. Second, the preoperative and intraoperative factors involved in skeletal muscle loss in the postoperative acute phase of ESCC were investigated. Then, whether skeletal muscle loss in the postoperative acute phase affected long-term prognosis was examined. The medical records of consecutive patients who underwent radical esophagectomy for ESCC between January 2010 and February 2015 were retrospectively reviewed; 72 cases were eligible for this study. The total psoas major muscle mass index (TPI) at the level of the third lumbar vertebra (L3) was measured using computed tomography (CT) before surgery and 3 days after surgery. The long-term prognosis was estimated by the Kaplan-Meier method and the multivariate logistic regression model.

**Results:**

There was already a significant reduction of TPI in the acute phase up to POD 3 after ESCC surgery in comparison with the preoperative baseline TPI (*P* < 0.001). The TPI reduction rate was significantly milder in cases with less blood loss during surgery and in cases that underwent thoracoscopic esophagectomy than in cases that underwent open esophagectomy. The 3-year overall survival rate was significantly different between the TPI reduction rate severe group and the TPI reduction rate mild group.

**Conclusion:**

Skeletal muscle loss occurred even in the postoperative acute phase. Furthermore, it is very significant that skeletal muscle loss in the postoperative acute phase of ESCC surgery is involved in the long-term prognosis.

## Background

Esophageal cancer is the sixth leading cause of cancer-related mortality worldwide, and squamous cell carcinoma is a common histological type of esophageal cancer in East Asian countries [1]. Advanced esophageal cancer (ESCC) is still a clinically challenging disease that requires a trimodal approach combining surgery, chemotherapy, and radiotherapy. To achieve it, the multidisciplinary team should have expertise in pathology, radiology, endoscopy, medical oncology, surgery, nursing, dietetics, and other relevant specialists as needed. Despite the advances of multimodality therapy, radical esophagectomy (subtotal esophagectomy with two/three-field lymph node dissection) remains the mainstream treatment for advanced resectable esophageal cancers [2]. Although the surgical technique and perioperative management for ESCC have developed, the postoperative survival rate of ESCC remains poor compared with other gastrointestinal cancers [3, 4]. Thus, specific prediction markers for the postoperative outcomes of ESCC have been sought for the purpose of contributing to a better prognosis.

Sarcopenia was defined as the loss of skeletal muscle volume, skeletal muscle strength, and physical function in the diagnostic criteria of the European Working Group on Sarcopenia in Older People [5]. Sarcopenia is classified into primary sarcopenia and secondary sarcopenia according to its cause. Primary sarcopenia is simply caused by aging, and secondary sarcopenia is caused by activity loss (disuse), disease, or malnutrition [5].

In gastrointestinal cancers, not only the inflammation and hypercatabolism caused by the cancer itself, but also the malnutrition caused by organic changes such as a passage disorder, are likely to lead to skeletal muscle loss as a secondary sarcopenia. Furthermore, cancer treatments, including surgery, are also considered to cause skeletal muscle loss due to malnutrition, disuse, and hypercatabolism due to the side effects of their invasiveness [6, 7].

Recently, there have been many studies on skeletal muscle loss during the perioperative phase, including in gastrointestinal cancers. However, most analyses were of sarcopenia before surgery using the index calculated by the standard formula, and they reported that it was a risk factor for postoperative complications and a poor long-term prognosis [8–13]. Regarding skeletal muscle loss after surgery, there have been a few studies of skeletal muscle loss concerned with postoperative outcomes only in cancers of organs other than the gastrointestinal tract [14]. On the other hand, there also have been reports that body composition changes after gastrointestinal cancer surgery, such as body weight loss (BWL) and skeletal muscle loss, were greater in the postoperative acute phase (around 1 week after surgery) than in the chronic phase (about 1 or more months after surgery) [14]. In view of the above, we hypothesized that skeletal muscle loss in the postoperative acute phase might be a new predictor for long-term prognosis, especially for highly invasive surgery such as ESCC surgery. However, to the best of our knowledge, no previous study has focused on the relationship between skeletal muscle loss in the postoperative acute phase and postoperative outcomes, including long-term prognosis.

In our university hospital, routine computed tomography (CT) has been performed on postoperative day (POD) 3 for all ESCC patients who underwent surgery to detect postoperative acute complications for more than 10 years. Thus, many CT image files of the postoperative very acute phase of ESCC patients are available in our hospital. Thus, it was possible to analyze skeletal muscle loss using them in comparison with baseline image files. In this study, the following items were retrospectively investigated. First, whether skeletal muscle loss occurred in the postoperative acute phase of ESCC was verified. Second, the preoperative and intraoperative factors involved in skeletal muscle loss in the postoperative acute phase of ESCC were investigated. Then, whether skeletal muscle loss in the postoperative acute phase affected the long-term prognosis was examined.

## Methods

### Patients

**Fig. 1:**
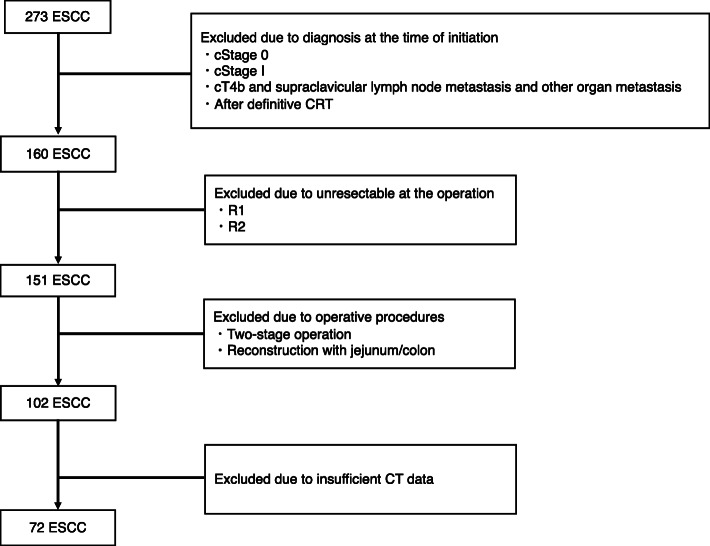
Case flow chart. cStage, clinical stage (UICC8th); CRT, chemoradiation; R, residual tumor

The medical records of consecutive patients who underwent radical esophagectomy (subtotal esophagectomy with two/three-field lymph node dissection) for ESCC at the Okayama University Hospital (Okayama, Japan) from January 2010 to February 2015 were retrospectively reviewed. A total of 273 ESCC cases without evidence of distant metastases underwent radical esophagectomy. To investigate advanced resectable ESCC, cases with clinical stage over II were selected, excluding those with T4b and supraclavicular lymph node metastasis and other organ metastasis at the time of treatment initiation. Four patients after definitive chemoradiotherapy were excluded. Nine patients with unresectable or residual cancer (R1/2) at operation were also excluded. To eliminate differences in surgical invasiveness, the operative procedure was limited to radical esophagectomy with gastric tube reconstruction; other procedures (two-stage operation, reconstruction with jejunum/colon) were excluded. Cases that underwent combined resection of other organs with cancer were also excluded. In addition, 30 cases were excluded because of insufficient CT data. Thus, 72 cases were eligible for this study (Fig. 1).

### Surgical procedures

Esophagectomy with two/three-field lymph node dissection was performed. We had been performing open esophagectomy (OE) until June 2011 when thoracoscopic esophagectomy in the prone position (TEPP) was introduced. After that, we have been performing TEPP as standard procedure. In this study, 22 cases underwent OE, and 50 cases underwent TEPP. We also investigated the relationship between those surgical procedures and skeletal muscle loss, which may be an invasive marker. Subsequently, abdominal lymphadenectomy and gastric conduit reconstruction were performed under laparotomy or hand-assisted laparoscopic surgery (HALS). The details of the surgical techniques of radical esophagectomy have been reported [15]. Data of the surgical procedure, operative time, amount of blood loss, and infusion volume were recorded as operative factors.

### Postoperative management

During this period, there was no change in postoperative care, including drugs administered (methylprednisolone, antibiotics, proton pump inhibitors, catecholamine). The patients were admitted to the intensive care unit (ICU) on mechanical ventilation after radical esophagectomy. The patients were extubated on POD 1 if they had no problems on chest X-ray, blood examinations, and bronchoscopy. On POD 3, CT was performed to detect postoperative acute complications. If there were no serious findings on CT, and the patient’s course was good, the patient could be discharged from the ICU.

### CT analysis

The preoperative baseline CT examination was taken within 1 week before surgery (at the time admission for surgery) for the cases with preoperative therapy or preoperative sarcopenia and taken within 1 month before surgery for the cases without both preoperative therapy and preoperative sarcopenia. The original purpose of the CT examination in the postoperative acute phase (POD 3) was to detect postoperative complications such as pneumonia, anastomotic leakage, and venous thrombosis for all patients who underwent ESCC surgery. Therefore, the changes in skeletal muscle mass could be analyzed using the CT images of the postoperative acute phase and preoperative baseline images before surgery.

### Measurement of skeletal muscle loss

**Fig. 2:**
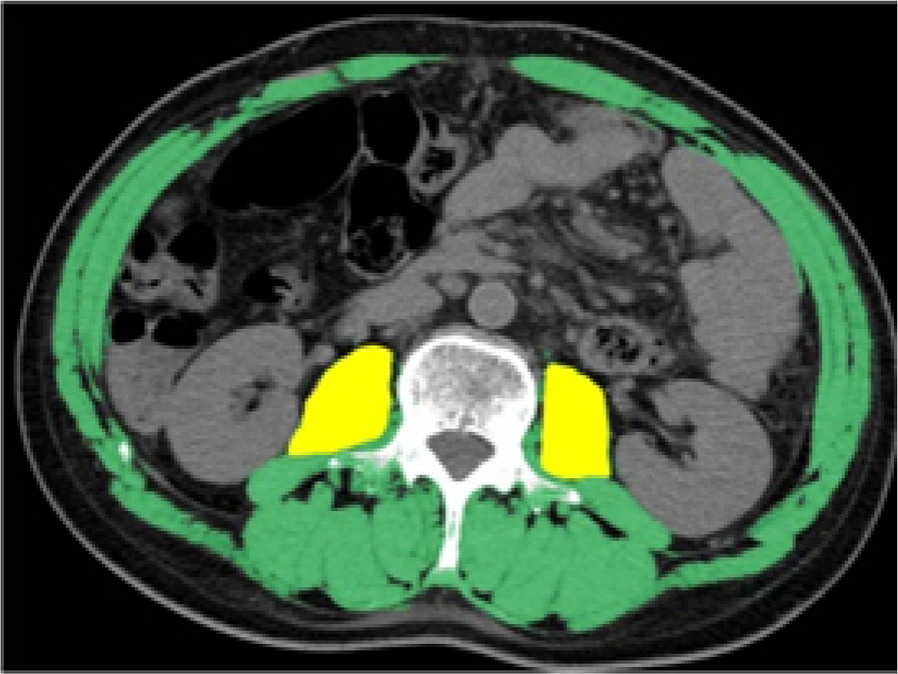
Measurement of skeletal muscle mass at the level of the third lumbar vertebra (L3) with CT images. Green field, total muscle mass; yellow field, psoas muscle mass

Following previously published reports about sarcopenia, skeletal muscle mass was measured at the level of the third lumbar vertebra (L3) with CT images. Although there are many reports using total skeletal muscle mass at the L3 level [11, 13, 16–20], only the psoas muscle was measured to as much as possible exclude the effects of edema in the postoperative acute phase [6]. The images were evaluated using a CT image analysis system (Synapse Vincent, Fujifilm Medical, Tokyo, Japan). The iliopsoas area at the L3 level was measured using Hounsfield units (HU) of − 29 to + 150 (Fig. 2). The quantity of skeletal muscle was evaluated with the total psoas major muscle mass index (TPI). The TPI was defined by normalizing the cross-sectional areas of the bilateral psoas major muscles for height (cm^2^/m^2^). Furthermore, the change in the skeletal muscle loss from the preoperative state to the postoperative acute phase was analyzed using the change of TPI. The TPI reduction rate was calculated using the formula (postoperative TPI-preoperative TPI)/preoperative TPI × 100.

### Analyzed parameters

Skeletal muscle loss in the acute phase after ESCC surgery was retrospectively analyzed. For that analysis, the median TPI reduction rate was defined as the cutoff value to divide patients into the TPI reduction rate mild group and the TPI reduction rate severe group. The preoperative factors (age, blood data, tumor factors, preoperative sarcopenia, etc.) and intraoperative factors (surgical procedure, blood loss, operative time, etc.) concerned with skeletal muscle loss in the acute phase after ESCC surgery were investigated. Furthermore, the relationship between skeletal muscle loss in the acute phase after ESCC surgery and long-term prognosis was investigated together with other clinicopathological parameters including preoperative sarcopenia.

### Statistical analysis

Clinicopathological factors were noted according to the Union for International Cancer Control (UICC) Tumor Nodes Metastasis (TNM) Classification of Malignant Tumors, 8th edition [21]. To evaluate the differences between the two groups, continuous variables were assessed using the Wilcoxon rank-sum test, and categorical variables were evaluated using Fisher’s exact test. Differences were considered significant when the *P* value was < 0.05. Kaplan-Meier analyses were also used to estimate the cumulative survival of patients. To identify the preoperative prognostic factors for 3-year survival, all variables with *P* < 0.05 on the univariate analysis were included in the multivariate logistic regression model. All analyses were performed using the JMP version 14 statistical analysis software (SAS Institute, Cary, NC, USA).

## Results

### Clinicopathological characteristics of patients with ESCC

Of the 72 ESCC cases, 65 were male, and 7 were female. Physical status according to the ASA classification was grade 1 or 2 in 65 cases and grade 3 in 7 cases. The presence of preoperative sarcopenia was defined according to the standard from Prado’s report [22], and the cutoff value of the skeletal muscle mass index was determined to be 52.4 for males and 38.5 for females. Patients whose values were less than or equal to the cutoff values were defined as having sarcopenia, and 64 cases were diagnosed with preoperative sarcopenia. Of the 72 cases, 55 received preoperative therapy, and 17 did not. Fifty-four cases received preoperative chemotherapy, and one case received preoperative chemoradiotherapy. The surgical procedure was OE with right thoracotomy in 22 cases and TEPP as minimally invasive surgery MIS in 50 cases. Half of the cases were diagnosed with pathological stage III or higher (Table 1).

**Table 1:** Patients’ characteristics

Characteristic	Number of patients (%) *n* = 72	
Age, y, median (IQR)	66 (43 - 83)	
Sex, n (%)		
Male	65 (90%)	
Female	7 (10%)	
ASA, n (%)		
1, 2	65 (90%)	
≥ 3	7 (10%)	
Preoperative sarcopenia^b^, n (%)		
Sarcopenic	64 (89%)	
Nonsarcopenic	8 (11%)	
Tumor location, n (%)		
Ce	4 (6%)	
Ut	13 (18%)	
Mt	30 (42%)	
Lt	23 (32%)	
Ae	2 (3%)	
Operative procedure, n (%)		
Open esophagectomy	22 (31%)	
Thoracoscopic esophagectomy	50 (69%)	
Preoperative therapy, n (%)		
None	17 (24%)	
Chemotherapy	54 (75%)	
Chemoradiotherapy	1 (1%)	
Postoperative therapy (including adjuvant therapy), n (%)		
No	22 (31%)	
Yes	50 (69%)	
pStage, n (%)		
< pStage III	36 (50%)	
≥ pStage III	36 (50%)	
TPI (cm^2^/m^2^), median (IQR)		
Preoperative TPI	4.63 (3.74 - 5.77)	*P* = 0.116^a^
Postoperative TPI	4.28 (3.62 - 5.28)	

*TPI* total psoas major muscle mass index, *pStage* pathological stage (UICC 8th), *ASA* the American Society of Anesthesia physical status, *IQR* interquartile range, ^a^Wilcoxon rank-sum test, ^b^According to the definition of Prado [22]

### Change of TPI in the acute phase after ESCC surgery

**Fig. 3:**
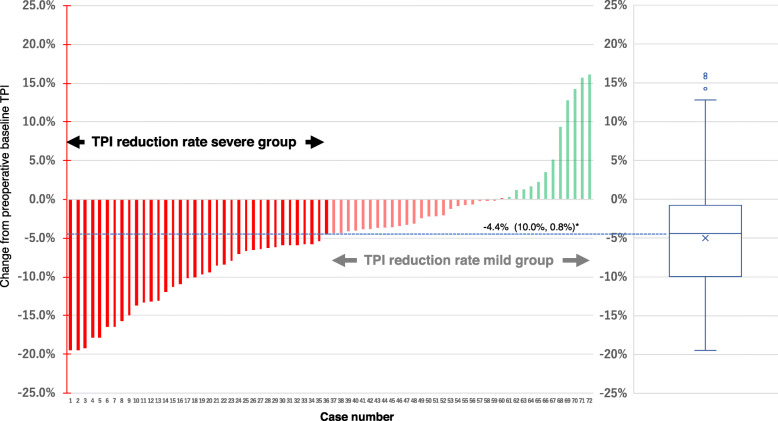
Change of TPI in the acute phase after ESCC surgery from preoperative baseline TPI (*n* = 72). TPI, total psoas major muscle mass index; *median (25%, 75%)

First, whether skeletal muscle loss occurred in the acute phase after ESCC surgery was examined. Out of the 72 cases, 59 cases’ (81.9%) TPI was decreased in the acute phase up to POD 3 after ESCC surgery, and the median TPI reduction rate was − 4.4%. There was already a significant change in of TPI in the acute phase up to POD 3 after ESCC surgery in comparison with the preoperative baseline TPI (*P* < 0.001) (Fig. 3).

### Associations between patient characteristics and skeletal muscle loss in ESCC surgery

The median TPI change rate (− 4.4%) was used as the cutoff value to divide patients into the TPI reduction rate mild group (*n* = 36) and the TPI reduction rate severe group (*n* = 36). The factors involved in skeletal muscle loss in the acute phase of ESCC surgery were examined. TPI reduction rate was significantly milder in cases of less blood loss during surgery (*P* = 0.0087) and in cases of TEPP than in cases of OE (*P* = 0.0407) (Table 2).

**Table 2:** Correlations between characteristics and sarcopenia in patients undergoing radical esophagectomy

Variable	Total cases	TPI reduction rate mild group	TPI reduction rate severe group	*P* value
Total cases, n (%)	72 (100)	36 (50%)	36 (50%)	-
Age at RE (y), median (IQR)	66 (43 - 83)	66 (55 - 83)	66 (44 - 82)	0.9380^a^
ASA, n (%)				0.6908^b^
1, 2	65 (90%)	33 (92%)	32 (89%)	
≥ 3	7 (10%)	3 (8%)	4 (11%)	
Preoperative sarcopenia^d^, n (%)				0.4533^b^
Sarcopenic	64 (89%)	33 (92%)	31 (86%)	
Nonsarcopenic	8 (11%)	3 (8%)	5 (14%)	
Tumor location, n (%)				0.5798^c^
Ce/Ut	17 (24%)	10 (28%)	7 (19%)	
Mt/Lt/Ae	55 (76%)	26 (72%)	29 (81%)	
pStage, n (%)				0.3450^b^
< pStage III	36 (50%)	21 (58%)	15 (42%)	
≥ pStage III	36 (50%)	15 (42%)	21 (58%)	
Preoperative therapy, n (%)				0.5675^c^
None	17 (24%)	8 (22%)	9 (25%)	
Chemotherapy	54 (75%)	28 (78%)	26 (72%)	
Chemoradiotherapy	1 (1%)	0 (0%)	1 (3%)	
Postoperative therapy, n (%)				0.5675^c^
None	17 (24%)	8 (22%)	9 (25%)	
Chemotherapy	51 (75%)	28 (78%)	26 (72%)	
Chemoradiotherapy	1 (1%)	0 (0%)	1 (3%)	
Operative procedure, n (%)				0.0407^b^
Open esophagectomy	22 (31%)	7 (19%)	15 (42%)	
Thoracoscopic esophagectomy	50 (69%)	29 (81%)	21 (58%)	
Blood loss (ml), median (IQR)	330 (150 - 510)	230 (110 - 405)	370 (255 - 723.75)	0.0087^a^
Operative time (min), median (IQR)	616.5 (553.5 - 698)	622 (540.75 - 728.5)	610.5 (559.25 - 676.5)	0.4885^a^

*TPI* total psoas major muscle mass index, *RE* radical esophagectomy, *IQR* interquartile range, *ASA* the American Society of Anesthesia physical status, *pStage* pathological stage (UICC 8th), ^a^Wilcoxon rank-sum test, ^b^Fisher’s exact test, ^c^Chi-square test, ^d^According to the definition of Prado [22]

### Associations between clinicopathological factors including the TPI reduction rate and long-term survival after ESCC surgery

**Fig. 4:**
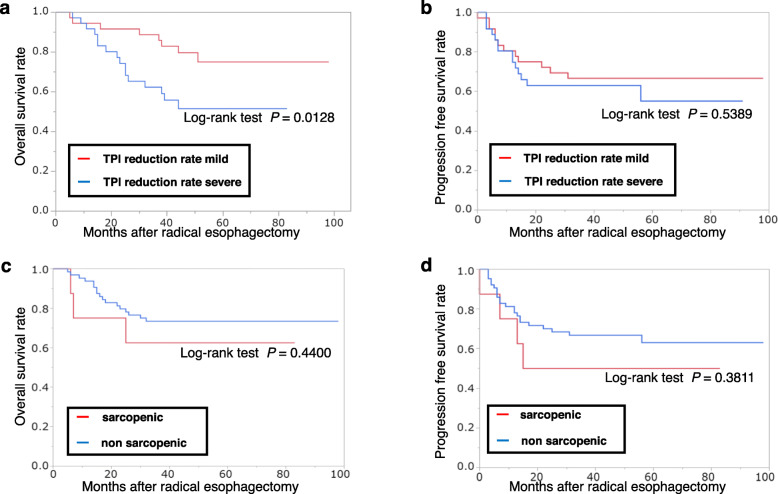
Overall survival curve of the groups with mild and severe reduction of the TPI (**a**). Progression-free survival curve of the groups with mild and severe reduction of the TPI. TPI, total psoas major muscle mass index (**b**). Overall survival curve of the groups with and without sarcopenia (**c**). Progression-free survival curve of the groups with and without sarcopenia (**d**)

The 3-year overall survival rate was significantly lower in the TPI reduction rate severe group than in the TPI reduction rate mild group (*P* = 0.0128). No significant difference between the two groups about progression-free survival (Fig. 4a, b). On the other hand, no significant difference between the two groups about with or without sarcopenia (Fig. 4c, d). Of the patients’ characteristics, surgical factors, and TPI reduction rate, the TPI reduction rate was an independent prognostic factor (*P* = 0.0296), along with pStage (*P* = 0.0039) and the presence of preoperative therapy (*P* = 0.0137) (Tables 3 and 4).

**Table 3:** Univariate logistic regression analysis of factors predicting overall survival in patients undergoing radical esophagectomy

Variable	*P* value	Odds ratio	95% CI
Pre-op Sarcopenia^a^	0.5259	0.60	0.13 - 3.19
TPI reduction rate severe group	0.0074*	4.43	1.47 - 15.3
Age ≥ 66 y	0.0942	2.57	0.86 - 8.86
≥ ASA 3	0.0109*	8.33	1.62 - 62.47
Pre-op Albumin < 3.6 g/dL	0.7550	1.33	0.17 - 7.46
No preoperative therapy	0.0492*	3.16	1.00 - 10.21
No postoperative therapy	0.5367	0.72	0.25 - 2.03
Pre-op BMI < 18.5 kg/m^2^	0.3262	0.47	0.07 - 2.00
Open or TEPP	0.1053	2.45	0.83 - 7.33
Open or HALS	0.2610	1.84	0.63 - 5.35
LN dissection field (2-field / 3-field)	0.5692	1.36	0.48 - 4.15
Blood loss ( > 330 ml)	0.4524	1.49	0.53 - 4.29
Operation time ( > 616 min)	1.0000	1.0000	0.35 - 2.83
≥ pStage III	0.0012*	6.40	2.02 - 24.87

*Pre-op* preoperative, *TPI* total psoas major muscle mass index, *ASA* the American Society of Anesthesia physical status, *BMI* Body mass index, *TEPP* Thoracoscopic esophagectomy in the prone position, *HALS* Hand-assisted laparoscopic surgery, *LN* lymph node, *pStage* pathological stage (UICC 8th), ^a^According to the definition of Prado [22], *p value <0.05

**Table 4:** Multivariate logistic regression analysis of factors predicting overall survival in patients undergoing radical esophagectomy

Variable	*P* value (Prob > ChiSq)	Odds ratio	95% CI
TPI reduction rate severe group	0.0296*	4.38	1.15 - 20.06
≥ ASA 3	0.0540	7.62	0.97 - 92.24
No preoperative therapy	0.0137*	7.07	1.48 - 42.09
≥ pStage III	0.0039*	7.65	1.86 - 43.05

*TPI* total psoas major muscle index, *ASA* the American Society of Anesthesia physical status, *pStage* Pathological stage (UICC 8th), *p value < 0.05

## Discussion

First, this study showed that skeletal muscle loss occurred in the very acute phase after ESCC surgery. Skeletal muscle loss has been known to easily occur in the postoperative period due to hypercatabolism, disuse, and malnutrition. In addition, there have been some reports of skeletal muscle loss in the postoperative relatively acute phase (7 to 10 days after surgery) in other gastrointestinal cancers [23, 24]. Even so, it was surprising that skeletal muscle loss already occurred on POD 3 after ESCC surgery.

Although the hypercatabolic phase induced by surgical invasion starts on POD 2 and lasts 3–8 days [25–28], and skeletal muscle loss due to disuse is also known to occur even after a few days of bed rest [29], malnutrition is not seen in the postoperative acute phase. Thus, it was thought that hypercatabolism and disuse might be the causes of skeletal muscle loss in that phase. According to the results of the present study, skeletal muscle loss in the postoperative acute phase might strongly reflect the hypercatabolism induced by surgical invasion because the TPI reduction rate was significantly related to intraoperative blood loss as a universal index of surgical invasion. On the other hand, the TPI reduction rate was significantly milder after TEPP than after OE. Although this might be one of the findings indicating the lesser invasiveness of TEPP, there was also another possibility: that TEPP as a painless procedure could maintain the postoperative patient’s physical activity and prevent the disuse followed by skeletal muscle loss. Based on the present study, it is difficult to clarify which was the main cause, hypercatabolism or disuse, and further investigation, such as bio-impedance analysis, is needed. However, in any case, the TPI reduction rate could be a useful parameter to identify the appropriate surgery for patients, and it might be an indicator showing the advantage of TEPP. Further studies are needed to determine whether thoracoscopic surgery is more useful than open surgery in esophageal cancer, and the results of ongoing clinical research of the Japan Clinical Oncology Group (JCOG 1409) are expected to clarify this point.

The core aspect of the present study is that, to the best of our knowledge, it is the first report showing the effect of skeletal muscle loss in the acute phase after surgery on the long-term prognosis of ESCC. Although there have been some reports that perioperative skeletal muscle loss was a prognostic predictor after surgeries for cancers including ESCC [8–14, 30], they examined skeletal muscle loss in the preoperative phase or the postoperative chronic phase (about 1 or more months after surgery). Surprisingly, in the present study, the TPI reduction rate on POD 3 after ESCC surgery was an independent long-term predictor of prognosis that was as significant as pathological stage and neoadjuvant treatment. Thus, skeletal muscle loss, even in the acute phase, might have a certain effect on the long-term prognosis.

Therefore, in order to improve patients’ prognosis after surgery for esophageal cancer, the early postoperative phase multidisciplinary team approaches, such as nutritional therapy and physical therapy, would be important to prevent skeletal muscle loss, similar to the preoperative intervention in previous reports [31–33]. On the other hand, preoperative sarcopenia was also pointed out in 89% of this study’s cohort. However, it was not found to be associated with the long-term prognosis despite previously published reports [30]. The reason for it might be that most cases in this study had sarcopenia, and there was no statistical difference with the TPI reduction rate about with or without sarcopenia. Furthermore, as an operative procedure, it might be a good option to select thoracoscopic surgery from the viewpoint of skeletal muscle loss using the TPI reduction rate as a new parameter of invasiveness or disuse.

Of course, there were several limitations in this study. This was a retrospective, small cohort study conducted at a single center only with CT image analysis. In addition, this study recruited more males than females. In the future, a large-scale investigation adding other analytical methods is needed.

## Conclusion

This is the first study that focused on the change of skeletal muscle volume between the preoperative phase and the postoperative early phase of esophageal cancer surgery. Skeletal muscle loss was an independent prognostic factor, along with pStage and the presence of preoperative therapy. It is very important that skeletal muscle loss in the postoperative acute phase was found to be related to long-term prognosis.

## Data Availability

The datasets used and/or analyzed during the current study are available from the corresponding author on reasonable request.

## Abbreviations

ESCC: Esophageal squamous cell carcinoma
TPI: Total psoas major muscle mass index
BWL: Body weight loss
CT: Computed tomography
POD: Postoperative day
OE: Open esophagectomy
TEPP: Thoracoscopic esophagectomy in the prone position
MIS: Minimally invasive surgery
HALS: Hand-assisted laparoscopic surgery
ICU: Intensive care unit
UICC: Union for International Cancer Control, TNM: Tumor Nodes Metastasis
